# Metabolomics Responses of Pearl Oysters (*Pinctada fucata martensii*) Fed a Formulated Diet Indoors and Cultured With Natural Diet Outdoors

**DOI:** 10.3389/fphys.2018.00944

**Published:** 2018-07-19

**Authors:** Chuangye Yang, Ruijuan Hao, Xiaodong Du, Yuewen Deng, Ruijiao Sun, Qingheng Wang

**Affiliations:** ^1^Fisheries College, Guangdong Ocean University, Zhanjiang, China; ^2^Pearl Breeding and Processing Engineering Technology Research Centre of Guangdong Province, Zhanjiang, China; ^3^Zhejiang Hengxing Food Co., Ltd., Jiaxing, China

**Keywords:** metabolomics, formulated diet, nutritional requirements, GC–MS, *Pinctada fucata martensii*

## Abstract

Natural disasters and environmental pollution are the main problems in traditional offshore cultivation. While culturing pearl oysters through industrial farming can avoid these problems, food availability in this case is limited. This study compares the metabolomics responses of pearl oysters, *Pinctada fucata martensii*, fed a formulated diet indoors with those of oysters cultured with natural diet outdoors by using a gas chromatography time-of-flight mass spectrometry (GC-TOF/MS)-based metabolomics approach. The animals were divided into two groups as follows: the experimental group (EG) was fed a formulated diet indoors and the control group (CG) was cultured with natural diet outdoors. After 45 days of feeding, the survival rate of EG was significantly higher than that of CG. The absolute growth rate (AGR) of the total weight of EG did not significantly differ from that of CG, but the AGRs of the shell length, shell height, and shell width of CG were significantly higher than those of EG. EG showed significantly higher amylase activities than CG, and the hexokinase and glucose-6-phosphate isomerase concentrations of the former were significantly lower than those of the latter. Metabolomics revealed 125 metabolites via mass spectrum matching with a spectral similarity value > 700 in the hepatopancreas, and 48 metabolites were considered to be significantly different between groups (VIP > 1 and *P* < 0.05). Pathway analysis results indicated that these significantly different metabolites were involved in 34 pathways. Further integrated key metabolic pathway analysis showed that, compared with CG, EG had lower capabilities for cysteine and methionine metabolism, sulfur metabolism, and starch and sucrose metabolism. This study demonstrated that the formulated diet could be an excellent substitute for natural diet; however, its nutrients were insufficient. Effective strategies should be developed to enhance the utilization of formulated diets.

## Introduction

*Pinctada fucata martensii* is the main pearl oyster species cultured for marine pearl production in China and Japan. In Southern China, production of this organism peaked in the 1990s with an annual yield of 20 tons (Yang et al., 2017a). However, pearl yield has steadily declined because of the slow growth and mass mortality of the cultured stock and environmental deterioration (Qiu et al., 2014). Thus, various methods, including new strain development (Deng et al., 2009; Deng Y.W et al., 2010), pearl culturing techniques (Deng C.M et al., 2010), and culture modes (Wang et al., 2016), have been applied to restore pearl yields. Traditional raft-culture models depend on natural microalgae and are highly susceptible to natural disasters and environmental pollution. These disadvantages can be avoided through industrial farming, where, unfortunately, the food demand is high and the available formulated diets for bivalves are limited.

Diet quality greatly affects an animal's performance. When animals are fed diets of different quality, alterations in key metabolic pathways are difficult to characterize by using traditional nutritional methods. Metabolomics is an effective omic technique to detect the overall complexity of and determine essential changes in diverse biological systems (Liu et al., 2016; Cappello et al., 2017, 2018). Low-molecular-weight metabolites, including lipids, sugars, and amino acids, can be quantified in biological samples by utilizing metabolomics approaches, such as ^1^H-nuclear magnetic resonance (NMR), gas chromatography–mass spectrometry (GC–MS), and liquid chromatography–mass spectrometry (LC–MS) (Ye et al., 2016; Maherizo et al., 2017; Venter et al., 2018). The identification and integrative analysis of these metabolites may enable the comprehensive characterization of metabolic mechanisms at the molecular and cellular levels under internal or external stimulating conditions. Among the metabolomics technologies currently available, gas chromatography time-of-flight mass spectrometry (GC–TOF/MS) is the most widely used because of its high resolution, high detection sensitivity, and numerous open-access spectral libraries (Sun et al., 2015; Collette et al., 2016; Hao et al., 2018). Thus, it can effectively assess the effects of food shortage (Tuffnail et al., 2009; Kullgren et al., 2010; Baumgarner and Cooper, 2012; Cipriano et al., 2015; Sheedy et al., 2016), nutrient supplementation (Andersen et al., 2014, 2015; Wagner et al., 2014), differences in nutrient levels (Jin et al., 2015), and dietary protein or lipid substitution (Abro et al., 2014; Cheng et al., 2016; Ma et al., 2017; Wei et al., 2017; Yang et al., 2018) in aquatic animals via metabolomics approaches.

The present study compares the metabolomics responses of pearl oysters *P. f. martensii* fed a formulated diet indoors with those of oysters cultured with natural diet outdoors by using a GC–TOF/MS-based metabolomics approach. The results can help enhance our understanding of the different mechanisms of *P. f. martensii* fed different diets and assist in the development of optimized nutritional requirements and feeding regimes.

## Materials and methods

### Experimental diet and procedures

The experimental diet was formulated according to the recommendations of previous research (Yang et al., 2017b, 2018); this diet had a typical proximate composition of 35% crude protein and 10% crude lipid and was stored at −20°C until use. Lipids were obtained from fish oils, while proteins were obtained from yeast powder and fish meal. The formulation and approximate composition of this diet were described by Yang et al. (2018). Pearl oysters [44.79 ± 1.25 mm in mean shell length (SL)] were assigned to the experimental group (EG) or control group (CG) randomly, and three replicates were prepared for both groups. Each replicate had 210 animals. The animals in EG were fed the formulated diet indoors, and the volume of water was 1,000 L. Diet doses for EG were specified according to a previous work (Wang et al., 2016), and the pearl oysters were fed every 6 h. Water was replaced daily at a volume of 300 L. CG was cultured with a natural diet outdoors. The experimental period lasted 45 days, and the following parameters were maintained: dissolved oxygen in water at 5.00 mg/L, water temperature at 20.5–22.5°C, and salinity at 30%. *P. f. martensii* is a lower invertebrate, and therefore, the study was not subject to ethical approval.

### Survival rate and growth rate

At the beginning and end of the experiment, the total number and growth performance of pearl oysters in each replicate were determined. SL, shell height (SH), and shell width (SW) were measured with a digital caliper (0.02 mm accuracy). Total weight (TW) was obtained with an electronic balance (0.01 g accuracy), and survival rates and absolute growth rates (AGRs) were calculated in accordance with the methods described by Yang et al. (2017b).

### Sample collection

At the end of the experiment, hepatopancreatic tissues from each animal were dissected, immediately kept in liquid nitrogen, and then stored at −80°C until analysis.

### Biochemical measurements

Eight hepatopancreatic tissues were collected from each replicate for biochemical measurements. Tissues were homogenized, and homogenates were centrifuged (10,000 × g for 20 min at 4°C) using a high-speed refrigerated centrifuge. The supernatant was transferred to new 2.0 mL tubes, and amylase activity was determined using commercial kits (Nanjing Jiancheng Bioengineering Research Institute, Nanjing, China) according to the manufacturer's instructions. Hexokinase (HK) and glucose-6-phosphate isomerase (G6PI) concentrations were also determined using commercial kits (Beijing Dongge Weiye Technology Co., Ltd., Beijing, China) according to the manufacturer's instructions. All assays were conducted within 24 h after extraction.

### Preparation for GC–MS analysis

Each replicate included 8 samples, and 24 samples were collected from each group for GC–MS analysis. Frozen hepatopancreas samples of approximately 100 mg were obtained from four individuals, transferred into 2 mL microcentrifuge tubes, and mixed with 0.5 mL of methanol extraction liquid (V_methanol_:V_chloroform_ = 3:1) and 20 μL of L-2-chlorophenylalanine (1 mg/mL stock in dH_2_O) as an internal standard. The samples were vortexed for 30 s, homogenized in a ball mill for 4 min at 45 Hz, subjected to ultrasound for 5 min, incubated in ice water, and centrifuged for 15 min at 12,000 rpm and 4 °C. The supernatant (0.45 mL) was transferred into fresh 2 mL GC–MS glass vials, dried in a vacuum concentrator without heating, added with 80 μL of methoxylamine hydrochloride (20 mg/mL in pyridine), and incubated for 30 min at 80°C. Approximately 100 μL of bis-(trimethylsilyl)-trifluoroacetamide reagent (1% trimethylchlorosilane, v/v) was added to the samples, which were then incubated for 2 h at 70°C.

### GC–MS analysis

GC–TOF/MS analysis was performed using an Agilent 7890 gas chromatograph system coupled to a Pegasus HT TOF/MS instrument. The system was equipped with a DB-5MS capillary column coated with 5% diphenyl cross-linked with 95% dimethylpolysiloxane (30 m × 250 μm inner diameter, 0.25 μm film thickness; J&W Scientific, Folsom, CA, USA). A 1 μL aliquot of the analyte was injected in split-less mode, and helium was used as the carrier gas. The front inlet purge flow was 3 mL min^−1^, and the gas flow rate through the column was 1 mL min^−1^. The initial temperature was maintained at 50°C for 1 min, raised to 310°C at a rate of 10°C min^−1^, and then maintained at 310°C for 8 min. The injection, transfer line, and ion source temperatures were 280, 270, and 220°C, respectively, and the energy was set to −70 eV in electron impact mode. Mass spectrometry data were acquired in full-scan mode over the *m*/*z* range of 50–500 at a rate of 20 spectra per second after a solvent delay of 6 min.

### Data analysis

Chroma TOF 4.3X software (LECO Corporation) and the LECO-Fiehn Rtx5 database were used for raw peak exaction, data baseline filtering and calibration, peak alignment, deconvolution analysis, peak identification, and peak area integration (Kind et al., 2009). The retention index (RI) was utilized to identify peaks, and an RI tolerance of 5,000 was set. The three-dimensional data obtained, including the peak number, sample name, and normalized peak area, were inputted into SIMCA14.1 (V14.1, MKS Data Analytics Solutions, Umea, Sweden) for principal component analysis (PCA) and orthogonal projections to latent structure–discriminate analysis (OPLS–DA). PCA showed the distribution of the original data. Supervised OPLS–DA was applied to obtain high-level group separation and enhance our understanding of the variables responsible for classification. The OPLS–DA model was employed with the first principal component of variable importance in the projection (VIP) values (VIP > 1) combined with Student's *t*-test (*P* < 0.05) to determine significantly different metabolites (SDMs) between the two groups. The fold change (FC) of each metabolite was calculated by comparing the mean values of the peak areas obtained from EG and CG. Commercial databases, including KEGG (http://www.genome.jp/kegg/), were utilized to search for the pathways of metabolites. MetaboAnalyst, a free Web-based tool that uses high-quality KEGG metabolic pathways as the backend knowledge base, was used for pathway analysis (http://www.metaboanalyst.ca).

The results of the growth and biochemical data were expressed as mean ± SEM, and significant differences (*P* < 0.05) among each variable were detected using *t*-test. All analyses were conducted using IBM SPSS Statistics 19 (IBM, USA).

## Results

### Growth, survival rate, and biochemical parameters in the hepatopancreas

**Figure 1:**
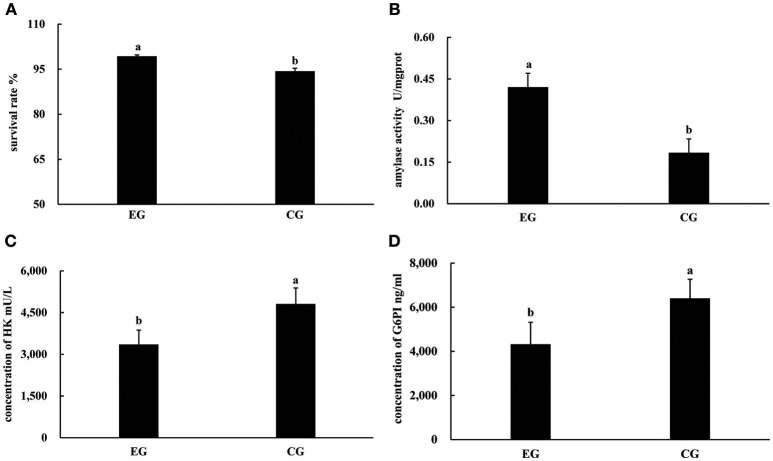
Survival rates, activities of amylase, concentrations of HK and G6PI in the hepatopancreas of *P. f. martensii* fed formulated diet indoors (EG) and cultured with a natural diet outdoors (CG). Means with the same letters are not significantly different (*P* > 0.05). **(A)** survival rates; **(B)** activities of amylase; **(C)** concentrations of HK; **(D)** concentrations of G6PI.

At the end of the experiment, the survival rates of pearl oysters in both groups ranged from 94.31 to 99.31%. The survival rate of CG was significantly lower than that of EG (*P* < 0.05, Figure 1), and the growth performance of the latter differed from that of the former (Table 1). Although the AGRs of the SL, SW, and SH of CG were significantly higher than those of EG (*P* < 0.05), the AGR of TW between EG and CG was not significantly different (*P* > 0.05). Pearl oysters in EG showed significantly higher amylase activity than those in CG (*P* < 0.05, Figure 1). By contrast, HK and G6PI concentrations in the hepatopancreas of the former were significantly lower than those of the latter (*P* < 0.05 Figure 1).

**Table 1:** Growth performance of pearl oyster (*P. f. martensii*) fed a formulated diet indoors (EG) and cultured with natural diet outdoors (CG).

	**EG**	**CG**
Shell length	3.90 ± 0.10*b*	4.42 ± 0.29*a*
Shell width	1.22 ± 0.22*b*	1.63 ± 0.39*a*
Shell height	3.97 ± 0.46*b*	4.26 ± 0.39*a*
Total weight	4.03 ± 0.32*a*	4.28 ± 0.36*a*

*Means with the same letters within a row are not significantly different (P >0.05)*.

### Total ion chromatograms (TICs) of hepatopancreas samples

**Figure 2:**
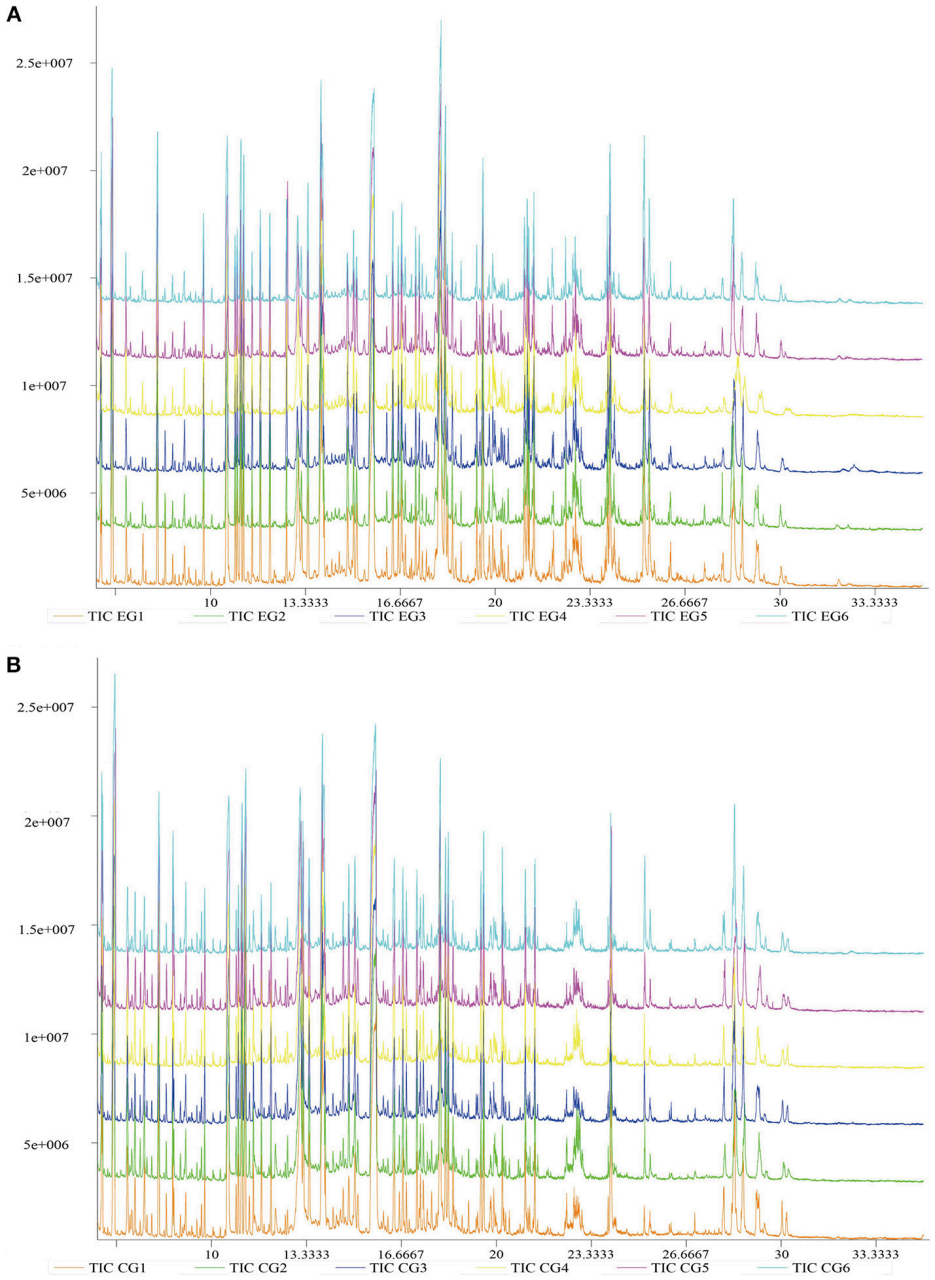
Typical GC–TOF/MS TICs of *P. f. martensii* hepatopancreas samples of EG and CG. The ordinate shows the relative mass abundance, and the abscissa shows the retention time. **(A)** Typical GC-TOF/MS TICs of EG; **(B)** Typical GC-TOF/MS TICs of CG.

Typical GC-TOF/MS TICs of *P. f. martensii* hepatopancreas samples from the two groups are shown in Figure 2. The standard deviation of the internal standard (L-2-chlorophenylalanine) retention time was 0.00255. The shape and number of peaks differed between EG and CG, and a total of 1,059 valid peaks were identified in the hepatopancreas. No drift was observed in any of the peaks that displayed a stable retention time. Thus, the TICs obtained by GC–TOF/MS could directly reflect differences in the metabolite profiles of EG and CG.

### PCA

**Figure 3:**
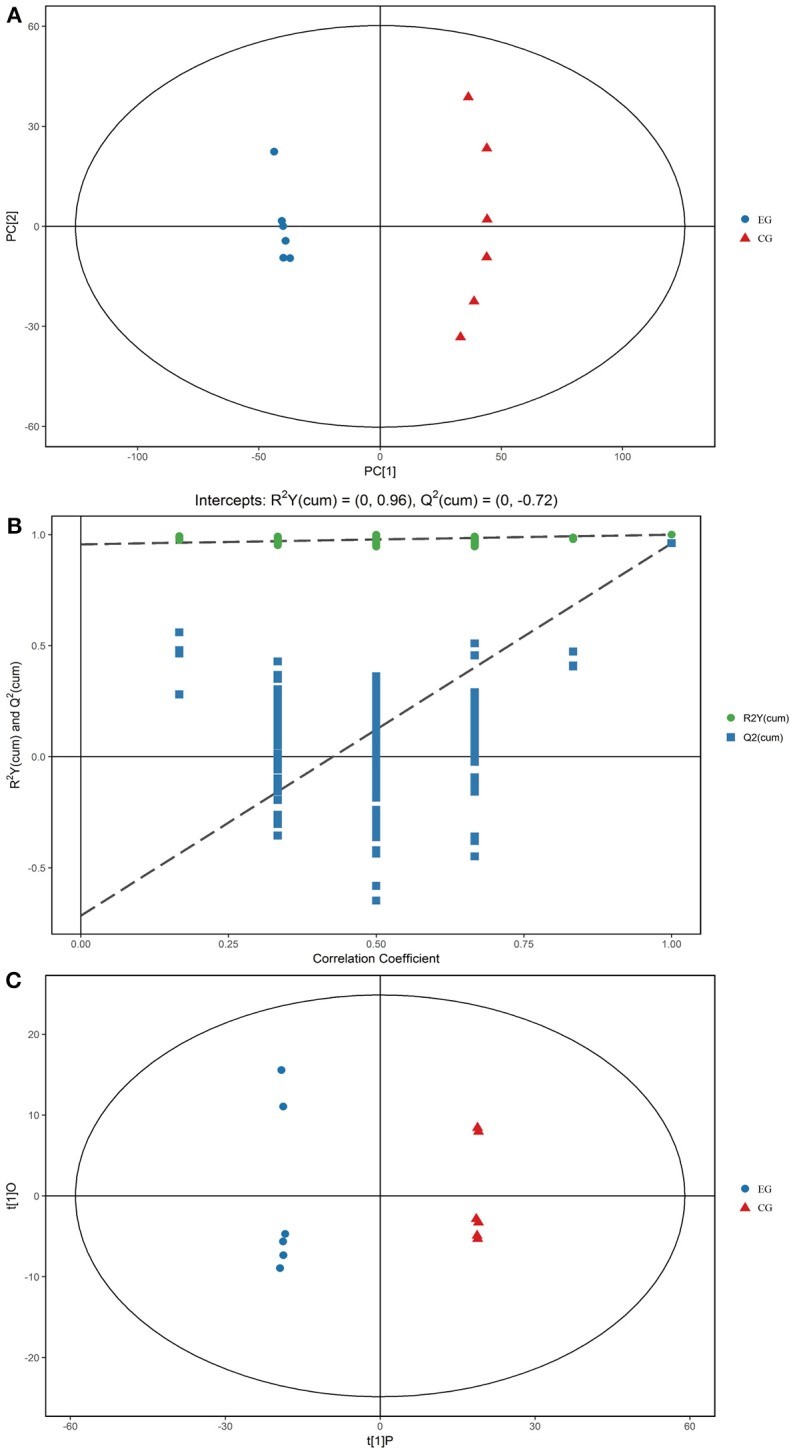
PCA score plots, OPLS-DA corresponding validation plots, and OPLS-DA score plots derived from the GC–TOF/MS metabolite profiles of the hepatopancreas of *P. f. martensii*. **(A)** PCA score plots; **(B)** OPLS-DA corresponding validation plots; **(C)** OPLS-DA score plots.

Gross changes in metabolic physiology are easily detectable by using the PCA of the entire set of measured analytes. The GC–TOF/MS metabolic profiles of the hepatopancreas between EG and CG showed markedly separated clusters in each score scatter plot of the PCA model (Figure 3A). Compared with that of CG, the R^2^X value of the PCA model representing the explained variance in the hepatopancreas of EG was 0.516. All of the samples in the score plots were within the 95% Hotelling's T-squared ellipse, thereby indicating no outlier present among the analyzed samples.

### OPLS-DA

OPLS-DA was conducted to enhance our understanding of different metabolic patterns. The parameters used to assess the quality of the OPLS-DA model in the hepatopancreas of EG could be represented by validation plots (Figure 3B). The parameters considered for classification from the software were R^2^X = 0.455, R^2^Y = 1, and Q^2^ = 0.961, all of which are stable and effective for fitness and prediction. The R^2^ and Q^2^ intercept values determined after 200 permutations were 0.96 and−0.72, respectively. The low values of the Q^2^ intercept indicate that the robustness of the models presents low risk of overfitting and reliability. Figure 3C displays the score scatter plots of the OPLS-DA model comparing EG and CG. All of the samples in the score plots were within the 95% Hotelling's T-squared ellipse, and clear separation and discrimination were found between the pair-wise groups. These findings indicate that the OPLS-DA model could be utilized to identify differences between the pair-wise groups.

### Metabolite identification and comparison

**Figure 4:**
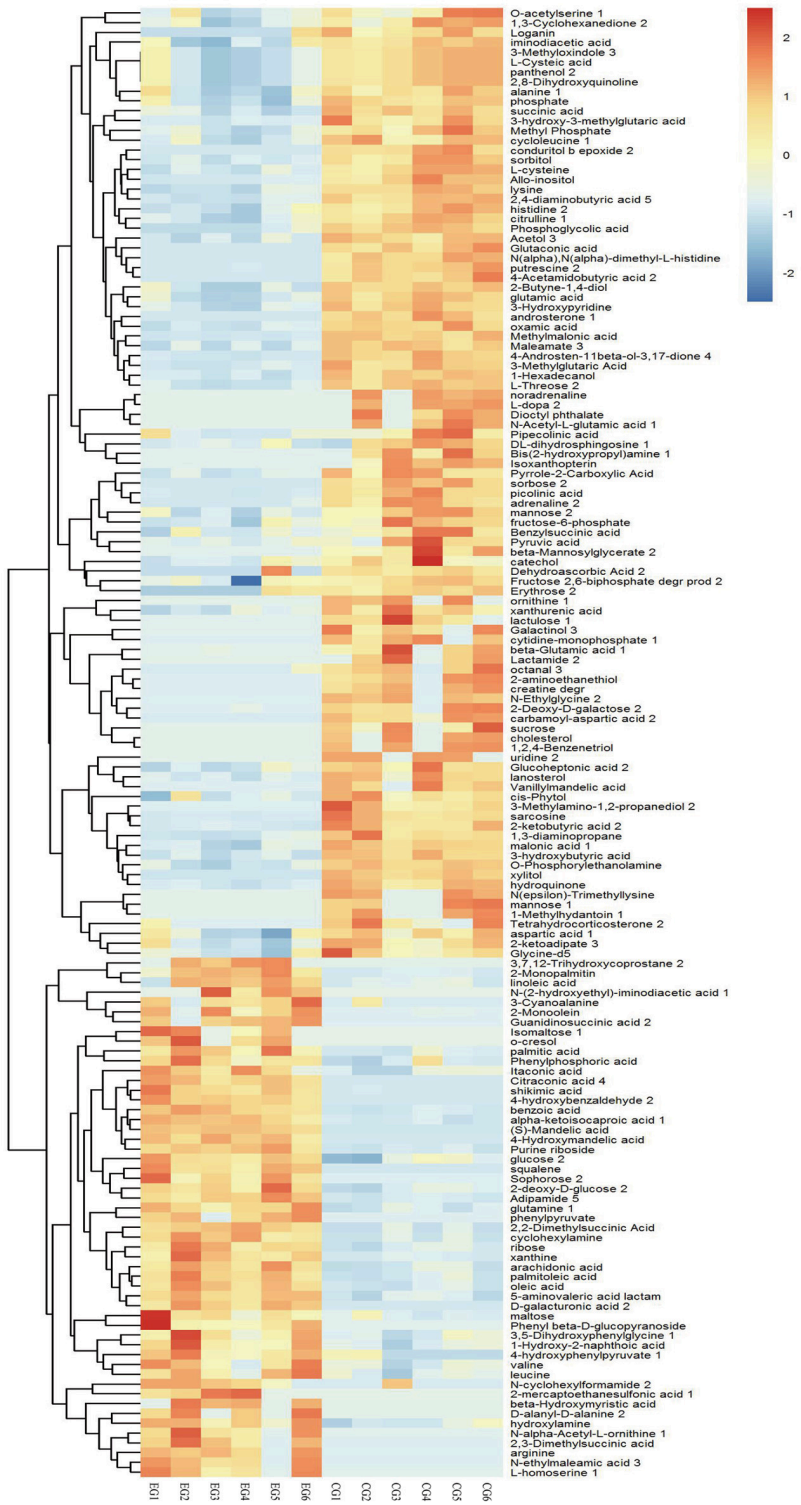
Hierarchical clustering analysis for SDMs. The relative metabolite level is depicted according to the color scale. Red indicates upregulation, while blue indicates downregulation.

A total of 1,015 peaks remained after filtering and denoising. The LECO/Fiehn Rtx5 Metabolomics Library suggested that most of the peaks detected were endogenous metabolites, although some may be by-product derivatives. A total of 562 metabolites were quantified, including 206 analytes (similarity value of > 0), and a total of 125 metabolites were identified by mass spectrum matching, with a spectral similarity value of > 700 (Supplemental Table 1). FC values were utilized to indicate specific variable quantities between EG and CG. Metabolite distribution could be visually divided into upregulated and downregulated metabolites. Among the 125 metabolites detected, 75 were downregulated in EG compared with CG (Supplemental Table 1). On the other hand, among 562 metabolites, 150 SDMs (VIP > 1 and *P* < 0.05) were determined in the hepatopancreas between EG and CG (Figure 4 and Supplemental Table 2). Among these 150 SDMs, 48 yielded a similarity value of > 700. These SDMs include 15 metabolites belonging to amino acids, peptides, and analogs, such as sarcosine, valine, alanine, and glutamic acid; 7 carbohydrate metabolites, including maltose, glucose, and mannose; 10 organic acids and derivatives, such as pyruvic acid and succinic acid; 11 lipid metabolites; and 5 other metabolites (Table 2). Among the 150 SDMs in the hepatopancreas, 54 yielded higher concentrations in EG than in CG (Figure 4 and Supplemental Table 2). By contrast, 96 metabolites in EG were significantly downregulated compared with those in CG.

**Table 2:** Identification of SDMs with similarity (Sim) > 700 in hepatopancreas between EG and CG groups.

**Metabolites**	**Sim**	**VIP**	***P*-value**	**FC**	**Log_2_FC**	**CT**
Palmitoleic acid	948	1.527	0.000	3.041	1.605	↑
Pyruvic acid	948	1.133	0.027	0.692	−0.531	↓
Oleic acid	947	1.559	0.000	2.804	1.487	↑
Sarcosine	945	1.603	0.000	0.020	−5.660	↓
Maltose	932	1.296	0.009	4.296	2.103	↑
Oleic acid	927	1.605	0.000	8.696	3.120	↑
Palmitic acid	926	1.396	0.002	1.361	0.445	↑
Glucose 2	923	1.306	0.001	1.501	0.586	↑
Valine	921	1.129	0.021	1.390	0.475	↑
O-Phosphorylethanolamine	921	1.561	0.000	0.640	−0.645	↓
Picolinic acid	918	1.593	0.000	0.201	−2.314	↓
Alanine 1	916	1.229	0.004	0.717	−0.481	↓
Phosphate	909	1.373	0.000	0.598	−0.742	↓
Glutamic acid	908	1.507	0.000	0.618	−0.693	↓
Cholesterol	908	1.162	0.026	0.000	−17.419	↓
Lysine	905	1.495	0.000	0.259	−1.950	↓
Xanthurenic acid	904	1.444	0.000	0.496	−1.012	↓
Beta-Mannosylglycerate 2	902	1.613	0.012	0.000	−21.091	↓
Glutamine 1	894	1.525	0.000	2.037	1.027	↑
Itaconic acid	888	1.302	0.002	1.635	0.709	↑
Mannose 2	882	1.376	0.001	0.581	−0.784	↓
Succinic acid	881	1.488	0.000	0.372	−1.425	↓
Isomaltose 1	880	1.113	0.049	344489.857	18.394	↑
Squalene	875	1.615	0.000	4231511.014	22.013	↑
Mannose 1	872	1.165	0.031	0.000	−18.522	↓
L-cysteine	871	1.503	0.000	0.317	−1.659	↓
Alpha-ketoisocaproic acid 1	863	1.535	0.000	11.302	3.499	↑
5-aminovaleric acid lactam	860	1.478	0.000	4.655	2.219	↑
citrulline 1	860	1.464	0.000	0.504	−0.989	↓
Ornithine 1	860	1.159	0.027	0.000	−25.208	↓
Methyl Phosphate	853	1.424	0.000	0.572	−0.807	↓
3-Hydroxypyridine	853	1.543	0.000	0.540	−0.890	↓
2-Monopalmitin	841	1.538	0.001	8.212	3.038	↑
Benzoic acid	818	1.596	0.000	2.423	1.277	↑
Putrescine 2	805	1.467	0.000	0.004	−7.796	↓
Uridine 2	804	1.162	0.025	0.000	−16.290	↓
Arachidonic acid	802	1.507	0.000	2.524	1.336	↑
2-Monopalmitin	801	1.616	0.000	4539096.110	22.114	↑
Sophorose 2	801	1.614	0.003	470699.180	18.844	↑
Ribose	800	1.501	0.000	2.332	1.221	↑
Pipecolinic acid	800	1.060	0.046	0.431	−1.213	↓
Galactinol 3	778	1.377	0.012	0.000	−18.881	↓
Conduritol b epoxide 2	758	1.615	0.000	0.000	−22.243	↓
Glucoheptonic acid 2	754	1.382	0.000	0.285	−1.810	↓
4-Hydroxymandelic acid	738	1.616	0.000	1213342.746	20.211	↑
histidine 2	718	1.385	0.000	0.354	−1.498	↓
2-Monoolein	703	1.176	0.012	66.257	6.050	↑
Leucine	703	1.236	0.004	1.756	0.812	↑

*FC represent Fold change; CT represent Change trend; ↑ and ↓ indicate that the metabolites were upregulated and downregulated in EG than CG, respectively*.

### Characterization and functional analysis of key metabolic pathways

**Figure 5:**
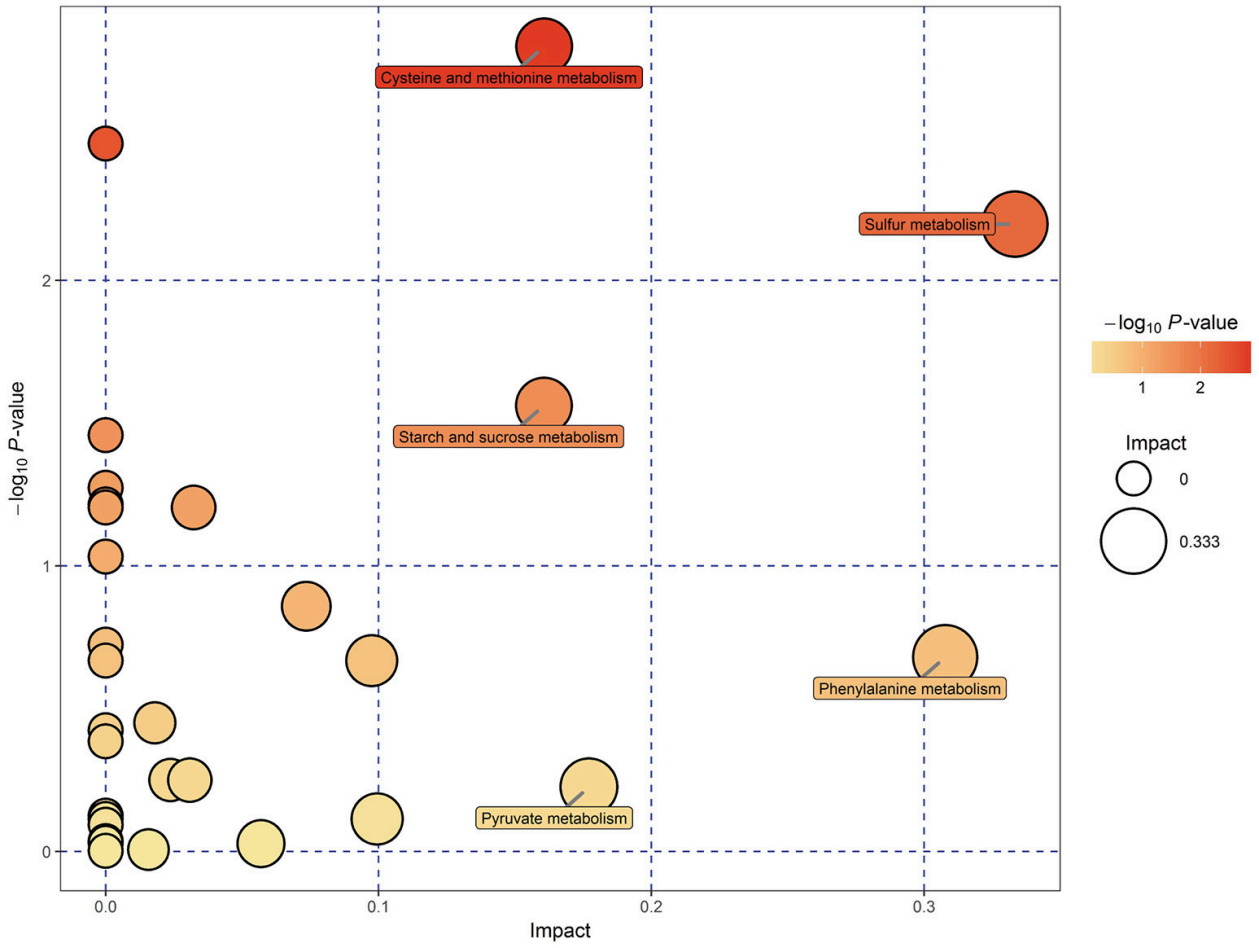
Metabolomic view map of significant metabolic pathways characterized in the hepatopancreas of *P. f. martensii* in EG and CG. This figure illustrates significantly changed pathways based on enrichment and topology analysis. The x-axis represents pathway enrichment, and the y-axis represents pathway impact. Large sizes and dark colors represent great pathway enrichment and high pathway impact values, respectively.

KEGG pathway analysis of SDMs was conducted by using MetaboAnalyst 3.0. Thirty-four pathways were found when the SDMs between EG and CG were imported into KEGG (Supplemental Table 3). However, only five of these pathways yielded an impact value of > 0.1, which is the relevance cut-off value, after the identified pathways were subjected to enrichment and pathway topology analyses (Supplemental Table 3). The impact values of sulfur metabolism, phenylalanine metabolism, pyruvate metabolism, cysteine and methionine metabolism, and starch and sucrose metabolism were 0.333, 0.308, 0.177, 0.161, and 0.161, respectively. Cysteine and methionine metabolism, sulfur metabolism, and starch and sucrose metabolism were characterized as relevant pathways based on P and impact values (Figure 5). In Table 3, the pyruvic acid, L-cysteine, L-cysteate, and *O*-acetyl-L-serine contents of EG were lower than those of CG, whereas the L-homoserine content was upregulated in cysteine and methionine metabolism. Similarly, the succinate, L-cysteine, and *O*-acetyl-L-serine contents of EG were lower than those of CG, whereas the L-homoserine content was upregulated in sulfur metabolism. The pyruvic acid, D-fructose-6-phosphate, and sucrose contents of EG were lower than those of CG, whereas the content of D-glucose, maltose, and isomaltose were upregulated in starch and sucrose metabolism.

**Table 3:** Metabolic pathways identified on the SDMs from the hepatopancreas between EG and CG groups.

**Metabolic pathway**	**SDMs**
Cysteine and methionine metabolism	(0.692) Pyruvic acid^*^ ↓
	(0.317) L-Cysteine ↓
	(347353.442) L-Homoserine ↑
	(0.753) L-Cysteate ↓
	(0.710) O-Acetyl-L-serine ↓
Starch and sucrose metabolism	(1.501) D-Glucose ↑
	(0.509) D-Fructose 6-phosphate ↓
	(0.026) Sucrose ↓
	(4.296) Maltose ↑
	(344489.857) Isomaltose ↑,
	(0.692) Pyruvic acid ↓
Sulfur metabolism	(0.372) Succinate ↓
	(0.317) L-Cysteine ↓
	(347353.442) L-Homoserine ↑
	(0.710) O-Acetyl-L-serine ↓
Phenylalanine metabolism	(0.692) Pyruvic acid ↓
	(0.372) Succinate ↓
	(477736.542) Phenylpyruvate ↑
	(2.423) Benzoate ↑
Pyruvate metabolism	(0.692) Pyruvic acid ↓
	(0.372) Succinate ↓

* *The number in the parentheses represents the value of Fold change (EG/CG); ↑ and ↓ indicate that the metabolites were upregulated and downregulated in EG than CG, respectively*.

## Discussion

Like many other bivalves, the pearl oyster *P. f. martensii* filters water-suspended particles, such as bacteria, organic debris, microalgae, and microzooplankton (Wang et al., 2016). However, microalgal culturing is labor intensive and difficult to control in large-scale production. Therefore, this process hardly satisfies food requirements for industrial farming development. In previous studies, formulated diets were shown to partially or completely replace microalgae in bivalves (Nevejan et al., 2009; Gui et al., 2016; Wang et al., 2016; Yang et al., 2017a,b). Nevertheless, commercially and biologically reliable artificial diets have yet to be prepared to substitute live microalgal feed for pearl oysters.

Yang et al. (2018) proved that yeast powder is a better protein source of formulated diet for pearl oysters than corn gluten via GC–TOF/MS-based metabolomics. Thus, to determine whether diets formulated with yeast powder as a major protein source achieves the ideal effects for breeding aquatics, comparison of pearl oysters fed formulated or natural diets in general is necessary. In the present study, the effects of a formulated diet on pearl oysters grown indoors were evaluated and compared with those of pearl oysters cultured with natural diet outdoors. At the end of the trials, the survival rate of EG was significantly higher than that of CG, which indicates that the formulated diet did not negatively affect *P. f. martensii*. The AGRs of EG for SL, SW, and SH of EG were lower than those of CG, but the two groups exhibited similar gains in TW. These results suggest that the formulated diet is an excellent substitute for natural diet.

Exploration of the mechanism behind the phenomenon is necessary to develop optimized nutritional requirements and feeding regimes and achieve optimum growth rates in aquaculture. Aquatic nutritional metabolomics research is an emerging field. To determine differences between the formulated and natural diets, we performed GC–TOF/MS-based metabolomics analysis, compared the metabolites, and detected changes in the hepatopancreas of pearl oysters between the two groups. Metabolomics is uniquely applicable to the assessment of metabolic responses to nutritional deficiencies or excesses. GC-TOF/MS-based metabolomics can detect low-molecular-weight metabolites and their intermediates and provide additional information on metabolic processes (Sun et al., 2015). In the present study, while 1,059 valid peaks were detected, the number of metabolites detected by GC–MS in plasma has been shown to vary among animal taxa in other studies (Kodama et al., 2014; Zaitsu et al., 2014). For example, 53 metabolites from the serum of tiger puffer fish (*Takifugu rubripes*) were obtained (Kodama et al., 2014), and 218 metabolites were found in the serum of cow (Sun et al., 2015). Although the metabolites in blood samples are secreted, excreted, or discarded from different animal tissues in response to physiological requirements or stress (Psychogios et al., 2011), the number of metabolites detected by GC–MS in serum is usually less than that in animal tissues. Ma et al. (2017) identified 222 peaks from the serum of *Eriocheir sinensis* but found that only 69 metabolites shared a similarity value of >700. In the present study, 562 peaks were detected in the hepatopancreas of the oysters, but only 125 of these peaks achieved a similarity value of >700. Among the 150 SDMs found between EG and CG, only 48 components showed a similarity value of >700. The number of metabolites with a similarity value of >700 in the present study was more than that (72 metabolites) obtained in the ovarian tissue of *Coilia nasus* (Xu et al., 2016).

**Figure 6:**
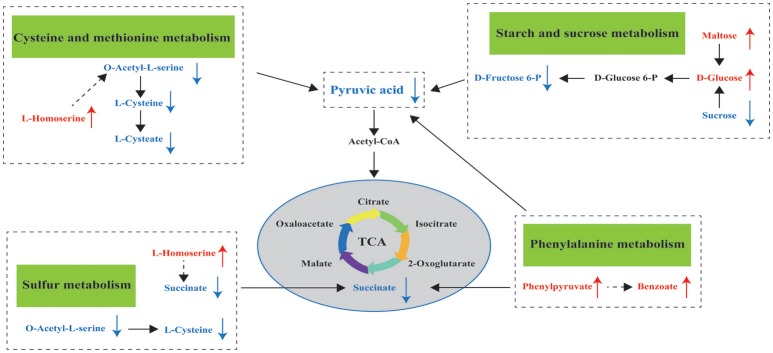
Hypothesized scheme of the metabolomics responses of pearl oysters *P. f. martensii* fed a formulated diet indoors or cultured with natural diet outdoors.

Metabolomics is a useful approach to obtain a whole-organism overview of affected pathways. The metabolic pathways identified from the SDMs represent the typical characteristics of dietary or medical intervention on organisms (Sun et al., 2015; Ma et al., 2017). The integrated key metabolic pathways are manually linked together based on the results of common key metabolic pathways and significantly changed pathways from different metabolites in the hepatopancreas. Three key metabolic pathways, including cysteine and methionine metabolism, sulfur metabolism, and starch and sucrose metabolism, were identified (Figure 6); these pathways are involved in amino acid metabolism, energy metabolism, and carbohydrate metabolism, respectively.

While L-homoserine levels in EG were significantly higher than those in CG, O-acetyl-L-serine and L-cysteine levels were downregulated in this group compared with those in CG. These observations suggest the effects of the formulated diet on cysteine and methionine metabolism and sulfur metabolism. L-homoserine is a nonessential amino acid that serves as the precursor of threonine and methionine; while it acts as an important functional amino acid, this acid does not participate in protein synthesis (Li et al., 2016). Low L-homoserine levels can improve growth traits and show similar threonine bioactivity in young chicks (Bryant et al., 2009). Zeng et al. (2017) reported that exogenous administration of glucose to fish upregulates L-homoserine content and greatly enhances their survival after infection. However, Bryant et al. (2009) confirmed that high levels of supplemental L-homoserine are noxious for chicks. Hao et al. (2018) surmised that high L-homoserine levels may affect glycine, serine, and threonine metabolism, as well as cysteine and methionine metabolism, thereby resulting in different growth performances in pearl oyster (*P. maxima*). Therefore, we propose that high L-homoserine levels in the hepatopancreas may influence amino acid metabolism, including cysteine and methionine metabolism and sulfur metabolism, and result in poorer growth performance of pearl oysters in EG compared with those in CG. However, the mechanisms underlying these modes of action have yet to be established and further investigated.

During starch and sucrose metabolism, the maltose, isomaltose, and D-glucose levels of EG were higher than those of CG. This finding proves that pearl oysters can break down α-starch in the formulated diet to produce glucose and is consistent with the higher amylase activity found in EG compared with that in CG. However, the glucose-6-phosphate, D-fructose-6-phosphate, and pyruvic acid levels in EG were downregulated (Supplemental Table 1) compared with those in CG. A decrease in levels of pyruvic acid, which is a product of glycolysis, implies a decline in glucose utilization (Rocha et al., 2011). The first step of intracellular glucose utilization is catalyzed by HK (Hall et al., 2009), and EG exhibits lower HK and G6PI concentrations compared with CG; such downregulation may result in the downregulation of D-fructose-6-phosphate and pyruvic acid. Therefore, the carbohydrate metabolism of EG is lower than that of CG, which could partly explain the poorer growth performance of pearl oysters in the former compared with those in the latter. The carbohydrate content of a diet can affect HK activity in aquatic animals (Enes et al., 2008; Moreira et al., 2008). For example, when dietary carbohydrate levels were maintained near the maximum tolerable level for metabolic utilization of carbohydrates by European sea bass juveniles, their liver HK did not increase vitality (Moreira et al., 2008). Thus, higher dietary carbohydrate levels may lead to lower HK and G6PI concentrations. This premise requires further investigations, and the effects of different dietary carbohydrate levels on pearl oyster should be studied.

β-oxidation is the catabolic process by which fatty acid molecules are broken down for energy production (Houten and Wanders, 2010). In the present study, some fatty acids such as 2-Monopalmitin (log_2_FC = 22.114), 2-Monoolein (log_2_FC = 6.050), oleic acid (log_2_FC = 8.696), and arachidonic acid (log_2_FC = 1.336) had very high FC values in EG2 (Table 2), attesting to the high fatty acid catabolic activity in EG2. Considering this, it can be hypothesized that the pearl oysters in EG2 required more energy via catabolism of fatty acids, which caused the poorer growth performance of pearl oysters in EG2 than those in CG. A similar phenomenon was also observed in farmed *Haliotis midae* (Venter et al., 2018). Squalene is the intermediate of cholesterol metabolism, and cholesterol is essential for membrane structure and hormone and steroid biosynthesis (Kalogeropoulos et al., 2010), such as cholecalciferol (VD3) biosynthesis, which can promote Ca^2+^ transport, absorption, and utilization, thereby regulating the biomineralization of pearl oyster shell. However, pearl oysters in EG achieved significantly higher squalene level (log_2_FC = 22.013) and significantly lower cholesterol level (log_2_FC = −17.419) than those in CG. This metabolic disorder could also cause a poor growth performance of pearl oysters in EG.

## Conclusions

Comparison of the growth performance and metabolic profiles of pearl oysters in EG and CG shows that the formulated diet could be an excellent substitute for natural diet. However, the formulated diet contains insufficient nutrients that may affect cysteine and methionine metabolism, sulfur metabolism, and starch and sucrose metabolism. Thus, modification of the dietary levels of these compounds in the formulated diet are required to achieve better growth performance, such as dietary carbohydrate level. Moreover, other strategies, such as formulating more effective diets and setting better feeding regimes, should be developed to enhance the utilization of formulated diets and improve pearl production and quality.

## Author contributions

YD, CY, RS, and XD designed the research. CY, RH, and YD conducted the research. CY and RH analyzed data. CY, RH, QW, XD, and YD contributed to the final writing of the paper. CY and RH wrote the manuscript. All authors have read and approved the final manuscript.

### Conflict of interest statement

The authors declare that the research was conducted in the absence of any commercial or financial relationships that could be construed as a potential conflict of interest.
