# Supplementary material for: Metabolomics Responses of Pearl Oysters (Pinctada fucata martensii) Fed a Formulated Diet Indoors and Cultured With Natural Diet Outdoors
Source: Front Physiol. 2018 Jul 19;9:944. doi: 10.3389/fphys.2018.00944 (PMC6060569; doi:10.3389/fphys.2018.00944)
Supplement: Supplementary file 1 [file Table_1.DOCX]

**Supplemental Table 1** Identification of metabolites with similarity (Sim) > 700 in hepatopancreas between the EG and CG groups.

| Peak | Sim | Mass | Mean-EG | Mean-CG | VIP | P-value | Fold change | Regulation |
| --- | --- | --- | --- | --- | --- | --- | --- | --- |
| pyruvic acid | 948 | 174 | 0.029 | 0.041 | 1.133 | 0.027 | 0.692 | ↓ |
| palmitoleic acid | 948 | 117 | 0.414 | 0.136 | 1.527 | 0.000 | 3.041 | ↑ |
| L-Allothreonine 1 | 947 | 219 | 0.206 | 0.422 | 0.922 | 0.070 | 0.488 | ↓ |
| oleic acid | 947 | 117 | 0.375 | 0.134 | 1.559 | 0.000 | 2.804 | ↑ |
| sarcosine | 945 | 116 | 0.067 | 3.405 | 1.603 | 0.000 | 0.020 | ↓ |
| Myristic Acid | 945 | 117 | 0.302 | 0.232 | 0.943 | 0.059 | 1.299 | ↑ |
| glycine 2 | 944 | 174 | 3.332 | 4.102 | 0.434 | 0.474 | 0.812 | ↓ |
| lactic acid | 943 | 117 | 0.047 | 0.273 | 0.156 | 0.021 | 0.172 | ↓ |
| uracil | 940 | 99 | 0.767 | 0.865 | 0.340 | 0.416 | 0.887 | ↓ |
| tyrosine 1 | 940 | 218 | 1.228 | 1.271 | 0.153 | 0.818 | 0.966 | ↓ |
| Isoleucine | 936 | 158 | 1.522 | 1.170 | 0.936 | 0.040 | 1.301 | ↑ |
| maltose | 932 | 360 | 0.088 | 0.020 | 1.296 | 0.009 | 4.296 | ↑ |
| phenylalanine 1 | 930 | 218 | 0.304 | 0.647 | 0.925 | 0.046 | 0.471 | ↓ |
| cholesterol | 930 | 445 | 0.001 | 0.000 | 0.911 | 0.082 | 73502.079 | ↑ |
| heptadecanoic acid | 927 | 117 | 0.165 | 0.176 | 0.695 | 0.842 | 0.936 | ↓ |
| oleic acid | 927 | 145 | 0.284 | 0.033 | 1.605 | 0.000 | 8.696 | ↑ |
| maltose | 927 | 361 | 0.343 | 0.140 | 0.325 | 0.062 | 2.440 | ↑ |
| palmitic acid | 926 | 132 | 1.394 | 1.024 | 1.396 | 0.002 | 1.361 | ↑ |
| serine 1 | 925 | 204 | 0.494 | 0.818 | 0.562 | 0.285 | 0.604 | ↓ |
| glucose 2 | 923 | 103 | 1.352 | 0.900 | 1.306 | 0.001 | 1.501 | ↑ |
| L-Malic acid | 922 | 233 | 0.372 | 0.428 | 0.792 | 0.112 | 0.868 | ↓ |
| valine | 921 | 144 | 2.104 | 1.514 | 1.129 | 0.021 | 1.390 | ↑ |
| O-Phosphorylethanolamine | 921 | 174 | 0.190 | 0.297 | 1.561 | 0.000 | 0.640 | ↓ |
| N-Acetyl-D-galactosamine 1 | 921 | 87 | 0.101 | 0.104 | 0.457 | 0.902 | 0.972 | ↓ |
| picolinic acid | 918 | 180 | 0.120 | 0.599 | 1.593 | 0.000 | 0.201 | ↓ |
| alanine 1 | 916 | 116 | 5.333 | 7.441 | 1.229 | 0.004 | 0.717 | ↓ |
| myo-inositol | 916 | 305 | 0.140 | 0.114 | 0.894 | 0.095 | 1.221 | ↑ |
| phosphate | 909 | 299 | 4.306 | 7.204 | 1.373 | 0.000 | 0.598 | ↓ |
| glutamic acid | 908 | 246 | 0.802 | 1.297 | 1.507 | 0.000 | 0.618 | ↓ |
| cholesterol | 908 | 339 | 0.000 | 0.003 | 1.162 | 0.026 | 0.000 | ↓ |
| lysine | 905 | 174 | 0.277 | 1.072 | 1.495 | 0.000 | 0.259 | ↓ |
| N-Methyl-DL-alanine | 904 | 130 | 0.077 | 0.064 | 0.787 | 0.088 | 1.213 | ↑ |
| xanthurenic acid | 904 | 406 | 0.100 | 0.202 | 1.444 | 0.000 | 0.496 | ↓ |
| beta-Mannosylglycerate 2 | 902 | 217 | 0.000 | 0.032 | 1.613 | 0.012 | 0.000 | ↓ |
| inosine | 902 | 451 | 0.005 | 0.004 | 0.547 | 0.498 | 1.235 | ↑ |
| stearic acid | 901 | 117 | 0.784 | 1.169 | 0.730 | 0.187 | 0.671 | ↓ |
| 2-hydroxypyridine | 896 | 152 | 0.563 | 0.708 | 0.655 | 0.566 | 0.796 | ↓ |
| glutamine 1 | 894 | 156 | 0.376 | 0.184 | 1.525 | 0.000 | 2.037 | ↑ |
| oxoproline | 891 | 156 | 7.787 | 13.532 | 0.523 | 0.016 | 0.575 | ↓ |
| Ciliatine | 891 | 397 | 0.163 | 0.139 | 0.488 | 0.445 | 1.172 | ↑ |
| beta-Alanine 2 | 890 | 174 | 0.841 | 0.423 | 0.358 | 0.115 | 1.987 | ↑ |
| Itaconic acid | 888 | 147 | 0.044 | 0.027 | 1.302 | 0.002 | 1.635 | ↑ |
| mannose 2 | 882 | 319 | 1.003 | 1.727 | 1.376 | 0.001 | 0.581 | ↓ |
| succinic acid | 881 | 147 | 3.515 | 9.439 | 1.488 | 0.000 | 0.372 | ↓ |
| Isomaltose 1 | 880 | 243 | 0.007 | 0.000 | 1.113 | 0.049 | 344489.857 | ↑ |
| squalene | 875 | 69 | 0.082 | 0.000 | 1.615 | 0.000 | 4231511.014 | ↑ |
| mannose 1 | 872 | 220 | 0.000 | 0.005 | 1.165 | 0.031 | 0.000 | ↓ |
| L-cysteine | 871 | 220 | 0.060 | 0.189 | 1.503 | 0.000 | 0.317 | ↓ |
| Ethanolamine | 870 | 174 | 0.073 | 0.095 | 0.497 | 0.223 | 0.773 | ↓ |
| proline | 869 | 142 | 2.192 | 2.449 | 0.478 | 0.374 | 0.895 | ↓ |
| D-(glycerol 1-phosphate) | 867 | 357 | 0.194 | 0.136 | 0.395 | 0.226 | 1.422 | ↑ |
| Sophorose 2 | 867 | 319 | 0.046 | 0.014 | 0.242 | 0.034 | 3.187 | ↑ |
| cholesterol | 865 | 243 | 0.003 | 0.003 | 0.266 | 0.881 | 0.891 | ↓ |
| alpha-ketoisocaproic acid 1 | 863 | 200 | 0.024 | 0.002 | 1.535 | 0.000 | 11.302 | ↑ |
| 5-aminovaleric acid lactam | 860 | 156 | 0.120 | 0.026 | 1.478 | 0.000 | 4.655 | ↑ |
| ornithine 1 | 860 | 142 | 0.000 | 0.562 | 1.159 | 0.027 | 0.000 | ↓ |
| citrulline 1 | 860 | 256 | 0.050 | 0.099 | 1.464 | 0.000 | 0.504 | ↓ |
| sulfuric acid | 857 | 227 | 0.000 | 0.014 | 0.959 | 0.089 | 0.000 | ↓ |
| 3-Hydroxypyridine | 853 | 152 | 0.079 | 0.146 | 1.543 | 0.000 | 0.540 | ↓ |
| Methyl Phosphate | 853 | 241 | 0.058 | 0.101 | 1.424 | 0.000 | 0.572 | ↓ |
| D-Glyceric acid | 853 | 189 | 0.032 | 0.019 | 0.555 | 0.027 | 1.677 | ↑ |
| lactose 1 | 853 | 204 | 0.000 | 0.001 | 0.548 | 0.363 | 0.000 | ↓ |
| inosine | 849 | 93 | 0.004 | 0.004 | 0.256 | 0.996 | 1.004 | ↑ |
| uridine 2 | 848 | 384 | 0.000 | 0.000 | 0.229 | 0.688 | 1.353 | ↑ |
| 2-Monopalmitin | 841 | 218 | 0.081 | 0.010 | 1.538 | 0.001 | 8.212 | ↑ |
| phenylethylamine | 836 | 174 | 0.030 | 0.054 | 0.345 | 0.172 | 0.564 | ↓ |
| uridine 2 | 835 | 217 | 0.009 | 0.002 | 0.003 | 0.506 | 3.762 | ↑ |
| palmitic acid | 830 | 357 | 0.001 | 0.001 | 0.017 | 0.969 | 0.974 | ↓ |
| arachidonic acid | 823 | 80 | 0.036 | 0.029 | 0.412 | 0.423 | 1.270 | ↑ |
| asparagine 4 | 821 | 243 | 0.096 | 0.101 | 0.453 | 0.826 | 0.944 | ↓ |
| N-Acetyl-D-galactosamine 1 | 821 | 333 | 0.005 | 0.007 | 0.706 | 0.330 | 0.733 | ↓ |
| glucose-6-phosphate 1 | 820 | 387 | 0.009 | 0.012 | 0.333 | 0.473 | 0.739 | ↓ |
| beta-Alanine 1 | 819 | 102 | 0.017 | 0.010 | 0.243 | 0.267 | 1.690 | ↑ |
| benzoic acid | 818 | 179 | 0.028 | 0.012 | 1.596 | 0.000 | 2.423 | ↑ |
| N-Acetyl-beta-D-mannosamine 3 | 812 | 202 | 0.030 | 0.031 | 0.454 | 0.918 | 0.977 | ↓ |
| glucose 1 | 808 | 462 | 0.000 | 0.004 | 0.759 | 0.006 | 0.006 | ↓ |
| N-Methyl-L-glutamic acid 3 | 806 | 98 | 0.050 | 0.206 | 0.173 | 0.014 | 0.242 | ↓ |
| putrescine 2 | 805 | 214 | 0.000 | 0.068 | 1.467 | 0.000 | 0.004 | ↓ |
| uridine 2 | 804 | 435 | 0.000 | 0.001 | 1.162 | 0.025 | 0.000 | ↓ |
| fumaric acid | 803 | 245 | 0.037 | 0.045 | 0.630 | 0.198 | 0.813 | ↓ |
| Aminomalonic acid | 802 | 218 | 0.152 | 0.281 | 0.348 | 0.114 | 0.540 | ↓ |
| arachidonic acid | 802 | 91 | 0.261 | 0.104 | 1.507 | 0.000 | 2.524 | ↑ |
| cis-gondoic acid | 802 | 290 | 0.001 | 0.000 | 0.903 | 0.090 | 34812.964 | ↑ |
| 2-Monopalmitin | 801 | 103 | 0.088 | 0.000 | 1.616 | 0.000 | 4539096.110 | ↑ |
| Sophorose 2 | 801 | 319 | 0.009 | 0.000 | 1.614 | 0.003 | 470699.180 | ↑ |
| Pipecolinic acid | 800 | 156 | 0.100 | 0.231 | 1.060 | 0.046 | 0.431 | ↓ |
| ribose | 800 | 307 | 0.020 | 0.009 | 1.501 | 0.000 | 2.332 | ↑ |
| fucose 2 | 800 | 117 | 0.056 | 0.069 | 0.487 | 0.348 | 0.821 | ↓ |
| 3,6-Anhydro-D-galactose 3 | 795 | 231 | 0.025 | 0.012 | 0.298 | 0.061 | 2.103 | ↑ |
| 1-Monopalmitin | 794 | 371 | 0.027 | 0.014 | 0.572 | 0.010 | 1.929 | ↑ |
| 5-Aminovaleric acid 1 | 791 | 174 | 0.134 | 0.149 | 0.392 | 0.402 | 0.902 | ↓ |
| adenosine | 788 | 236 | 0.000 | 0.002 | 0.955 | 0.084 | 0.000 | ↓ |
| alpha-Aminoadipic acid | 779 | 260 | 0.015 | 0.030 | 0.319 | 0.079 | 0.513 | ↓ |
| Galactinol 3 | 778 | 204 | 0.000 | 0.007 | 1.377 | 0.012 | 0.000 | ↓ |
| alpha-ketoglutaric acid | 772 | 198 | 0.010 | 0.019 | 1.031 | 0.053 | 0.505 | ↓ |
| Monostearin | 771 | 399 | 0.005 | 0.007 | 0.712 | 0.172 | 0.659 | ↓ |
| 3-Hydroxypropionic acid 1 | 768 | 177 | 0.000 | 0.001 | 0.854 | 0.034 | 0.143 | ↓ |
| guanosine | 766 | 352 | 0.001 | 0.000 | 0.909 | 0.167 | 37649.632 | ↑ |
| tyramine | 764 | 174 | 0.000 | 0.048 | 0.956 | 0.076 | 0.000 | ↓ |
| Phytol | 762 | 143 | 0.000 | 0.007 | 0.956 | 0.084 | 0.000 | ↓ |
| conduritol b epoxide 2 | 758 | 191 | 0.000 | 0.072 | 1.615 | 0.000 | 0.000 | ↓ |
| oxalic acid | 757 | 147 | 0.005 | 0.011 | 0.627 | 0.005 | 0.452 | ↓ |
| 3-Aminoisobutyric acid 2 | 755 | 102 | 0.033 | 0.035 | 0.426 | 0.395 | 0.929 | ↓ |
| Zymosterol 1 | 755 | 69 | 0.788 | 1.208 | 0.531 | 0.094 | 0.653 | ↓ |
| Glucoheptonic acid 2 | 754 | 217 | 0.397 | 1.392 | 1.382 | 0.000 | 0.285 | ↓ |
| citric acid | 753 | 273 | 0.325 | 0.262 | 0.940 | 0.059 | 1.243 | ↑ |
| thymidine 1 | 752 | 170 | 0.020 | 0.008 | 0.607 | 0.075 | 2.566 | ↑ |
| 1,5-Anhydroglucitol | 748 | 217 | 0.095 | 0.084 | 0.623 | 0.238 | 1.130 | ↑ |
| Stigmasterol | 745 | 386 | 0.022 | 0.039 | 0.358 | 0.151 | 0.558 | ↓ |
| Glucose-1-phosphate | 742 | 217 | 0.034 | 0.038 | 0.016 | 0.673 | 0.880 | ↓ |
| D-Arabitol | 739 | 103 | 0.015 | 0.019 | 0.487 | 0.408 | 0.824 | ↓ |
| 4-Hydroxymandelic acid | 738 | 267 | 0.023 | 0.000 | 1.616 | 0.000 | 1213342.746 | ↑ |
| thymine | 737 | 113 | 0.017 | 0.014 | 0.038 | 0.643 | 1.188 | ↑ |
| Glutaric Acid | 735 | 261 | 0.002 | 0.003 | 0.563 | 0.196 | 0.527 | ↓ |
| pentadecanoic acid | 732 | 132 | 0.039 | 0.039 | 0.117 | 0.867 | 1.017 | ↑ |
| Threonic acid | 730 | 292 | 0.007 | 0.006 | 0.272 | 0.655 | 1.212 | ↑ |
| pantothenic acid | 729 | 291 | 0.003 | 0.004 | 0.390 | 0.453 | 0.800 | ↓ |
| hypoxanthine 1 | 727 | 265 | 0.016 | 0.037 | 1.083 | 0.237 | 0.428 | ↓ |
| inosine 5'-monophosphate | 724 | 315 | 0.002 | 0.008 | 0.401 | 0.122 | 0.291 | ↓ |
| histidine 2 | 718 | 154 | 0.097 | 0.275 | 1.385 | 0.000 | 0.354 | ↓ |
| trans-4-hydroxy-L-proline 2 | 713 | 230 | 0.429 | 0.946 | 0.757 | 0.012 | 0.454 | ↓ |
| Neohesperidin | 713 | 273 | 0.000 | 0.001 | 1.067 | 0.082 | 0.253 | ↓ |
| leucine | 703 | 158 | 2.250 | 1.281 | 1.236 | 0.004 | 1.756 | ↑ |
| 2-Monoolein | 703 | 201 | 0.005 | 0.000 | 1.176 | 0.012 | 66.257 | ↑ |
| Lactobionic Acid 1 | 700 | 218 | 0.052 | 0.002 | 0.254 | 0.089 | 27.162 | ↑ |

↑ and ↓ indicate that the metabolites were upregulated and downregulated in EG than CG, respectively.
