# Supplementary material for: Metabolomics Responses of Pearl Oysters (Pinctada fucata martensii) Fed a Formulated Diet Indoors and Cultured With Natural Diet Outdoors
Source: Front Physiol. 2018 Jul 19;9:944. doi: 10.3389/fphys.2018.00944 (PMC6060569; doi:10.3389/fphys.2018.00944)
Supplement: Supplementary file 2 [file Table_2.DOCX]

**Supplemental Table 2** Identification of SDMs for group EG vs. CG

| Metabolites | Sim | Mass | VIP | p-value | Fold change |
| --- | --- | --- | --- | --- | --- |
| Pyruvic acid | 948 | 174 | 1.133 | 0.027 | 0.692 |
| palmitoleic acid | 948 | 117 | 1.527 | 0.000 | 3.041 |
| oleic acid | 947 | 117 | 1.559 | 0.000 | 2.804 |
| Sarcosine | 945 | 116 | 1.603 | 0.000 | 0.020 |
| Maltose | 932 | 360 | 1.296 | 0.009 | 4.296 |
| oleic acid | 927 | 145 | 1.605 | 0.000 | 8.696 |
| palmitic acid | 926 | 132 | 1.396 | 0.002 | 1.361 |
| glucose 2 | 923 | 103 | 1.306 | 0.001 | 1.501 |
| Valine | 921 | 144 | 1.129 | 0.021 | 1.390 |
| O-Phosphorylethanolamine | 921 | 174 | 1.561 | 0.000 | 0.640 |
| picolinic acid | 918 | 180 | 1.593 | 0.000 | 0.201 |
| alanine 1 | 916 | 116 | 1.229 | 0.004 | 0.717 |
| phosphate | 909 | 299 | 1.373 | 0.000 | 0.598 |
| glutamic acid | 908 | 246 | 1.507 | 0.000 | 0.618 |
| cholesterol | 908 | 339 | 1.162 | 0.026 | 0.000 |
| Lysine | 905 | 174 | 1.495 | 0.000 | 0.259 |
| xanthurenic acid | 904 | 406 | 1.444 | 0.000 | 0.496 |
| beta-Mannosylglycerate 2 | 902 | 217 | 1.613 | 0.012 | 0.000 |
| glutamine 1 | 894 | 156 | 1.525 | 0.000 | 2.037 |
| Itaconic acid | 888 | 147 | 1.302 | 0.002 | 1.635 |
| mannose 2 | 882 | 319 | 1.376 | 0.001 | 0.581 |
| succinic acid | 881 | 147 | 1.488 | 0.000 | 0.372 |
| Isomaltose 1 | 880 | 243 | 1.113 | 0.049 | 344489.857 |
| Squalene | 875 | 69 | 1.615 | 0.000 | 4231511.014 |
| mannose 1 | 872 | 220 | 1.165 | 0.031 | 0.000 |
| L-cysteine | 871 | 220 | 1.503 | 0.000 | 0.317 |
| alpha-ketoisocaproic acid 1 | 863 | 200 | 1.535 | 0.000 | 11.302 |
| 5-aminovaleric acid lactam | 860 | 156 | 1.478 | 0.000 | 4.655 |
| ornithine 1 | 860 | 142 | 1.159 | 0.027 | 0.000 |
| citrulline 1 | 860 | 256 | 1.464 | 0.000 | 0.504 |
| 3-Hydroxypyridine | 853 | 152 | 1.543 | 0.000 | 0.540 |
| Methyl Phosphate | 853 | 241 | 1.424 | 0.000 | 0.572 |
| 2-Monopalmitin | 841 | 218 | 1.538 | 0.001 | 8.212 |
| benzoic acid | 818 | 179 | 1.596 | 0.000 | 2.423 |
| putrescine 2 | 805 | 214 | 1.467 | 0.000 | 0.004 |
| uridine 2 | 804 | 435 | 1.162 | 0.025 | 0.000 |
| arachidonic acid | 802 | 91 | 1.507 | 0.000 | 2.524 |
| 2-Monopalmitin | 801 | 103 | 1.616 | 0.000 | 4539096.110 |
| Sophorose 2 | 801 | 319 | 1.614 | 0.003 | 470699.180 |
| Pipecolinic acid | 800 | 156 | 1.060 | 0.046 | 0.431 |
| Ribose | 800 | 307 | 1.501 | 0.000 | 2.332 |
| Galactinol 3 | 778 | 204 | 1.377 | 0.012 | 0.000 |
| conduritol b epoxide 2 | 758 | 191 | 1.615 | 0.000 | 0.000 |
| Glucoheptonic acid 2 | 754 | 217 | 1.382 | 0.000 | 0.285 |
| 4-Hydroxymandelic acid | 738 | 267 | 1.616 | 0.000 | 1213342.746 |
| histidine 2 | 718 | 154 | 1.385 | 0.000 | 0.354 |
| Leucine | 703 | 158 | 1.236 | 0.004 | 1.756 |
| 2-Monoolein | 703 | 201 | 1.176 | 0.012 | 66.257 |
| Allo-inositol | 686 | 217 | 1.573 | 0.000 | 0.120 |
| Fructose 2,6-biphosphate degr prod 2 | 679 | 227 | 1.114 | 0.008 | 0.848 |
| Phenyl beta-D-glucopyranoside | 677 | 114 | 1.613 | 0.016 | 422608.219 |
| Xylitol | 673 | 307 | 1.616 | 0.000 | 0.000 |
| iminodiacetic acid | 672 | 232 | 1.292 | 0.001 | 0.780 |
| beta-Glutamic acid 1 | 660 | 232 | 1.145 | 0.020 | 0.018 |
| lactulose 1 | 659 | 73 | 1.374 | 0.014 | 0.000 |
| fructose-6-phosphate | 657 | 315 | 1.285 | 0.002 | 0.509 |
| Xanthine | 653 | 181 | 1.560 | 0.001 | 11.202 |
| androsterone 1 | 649 | 134 | 1.615 | 0.000 | 0.000 |
| linoleic acid | 636 | 337 | 1.184 | 0.009 | 2.333 |
| N-Ethylglycine 2 | 624 | 130 | 1.380 | 0.004 | 0.000 |
| (S)-Mandelic acid | 622 | 179 | 1.616 | 0.000 | 1391051.368 |
| Pyrrole-2-Carboxylic Acid | 620 | 240 | 1.504 | 0.001 | 0.357 |
| Methylmalonic acid | 619 | 231 | 1.606 | 0.000 | 0.122 |
| 1,3-diaminopropane | 602 | 174 | 1.527 | 0.000 | 0.222 |
| 1-Hexadecanol | 599 | 299 | 1.584 | 0.000 | 0.307 |
| Sorbitol | 594 | 159 | 1.534 | 0.000 | 0.418 |
| 2-aminoethanethiol | 584 | 174 | 1.380 | 0.007 | 0.000 |
| phenylpyruvate | 581 | 141 | 1.343 | 0.005 | 477736.542 |
| Citraconic acid 4 | 576 | 235 | 1.615 | 0.000 | 145226.301 |
| 3-Methylamino-1,2-propanediol 2 | 575 | 190 | 1.615 | 0.003 | 0.000 |
| Sucrose | 574 | 218 | 1.379 | 0.026 | 0.000 |
| cytidine-monophosphate 1 | 573 | 215 | 1.378 | 0.007 | 0.000 |
| shikimic acid | 566 | 204 | 1.606 | 0.000 | 18.388 |
| 2,2-Dimethylsuccinic Acid | 562 | 261 | 1.239 | 0.000 | 13.161 |
| malonic acid 1 | 538 | 147 | 1.533 | 0.000 | 0.535 |
| oxamic acid | 536 | 147 | 1.575 | 0.000 | 0.355 |
| Bis(2-hydroxypropyl)amine 1 | 533 | 232 | 1.374 | 0.011 | 0.000 |
| 3,7,12-Trihydroxycoprostane 2 | 530 | 67 | 1.129 | 0.027 | 492183.759 |
| Loganin | 525 | 129 | 1.364 | 0.002 | 0.169 |
| O-acetylserine 1 | 523 | 174 | 1.089 | 0.022 | 0.710 |
| Purine riboside | 523 | 218 | 1.563 | 0.000 | 12.382 |
| DL-dihydrosphingosine 1 | 522 | 205 | 1.098 | 0.008 | 0.565 |
| D-galacturonic acid 2 | 514 | 292 | 1.615 | 0.000 | 157136.241 |
| Tetrahydrocorticosterone 2 | 512 | 243 | 1.100 | 0.022 | 0.117 |
| 1,2,4-Benzenetriol | 508 | 239 | 1.161 | 0.026 | 0.000 |
| 2-Butyne-1,4-diol | 499 | 217 | 1.505 | 0.000 | 0.613 |
| 4-Androsten-11beta-ol-3,17-dione 4 | 492 | 97 | 1.602 | 0.000 | 0.215 |
| o-cresol | 491 | 180 | 1.108 | 0.035 | 21900.910 |
| Lactamide 2 | 490 | 156 | 1.166 | 0.032 | 0.000 |
| 3-Methylglutaric Acid | 482 | 172 | 1.579 | 0.000 | 0.192 |
| Phosphoglycolic acid | 478 | 299 | 1.235 | 0.000 | 0.088 |
| 2-Deoxy-D-galactose 2 | 467 | 217 | 1.162 | 0.008 | 0.017 |
| 3-Cyanoalanine | 465 | 141 | 1.078 | 0.010 | 7.351 |
| 2-deoxy-D-glucose 2 | 461 | 292 | 1.483 | 0.000 | 2.355 |
| 2-ketobutyric acid 2 | 460 | 89 | 1.577 | 0.000 | 0.206 |
| Maleamate 3 | 459 | 58 | 1.041 | 0.000 | 0.116 |
| L-Threose 2 | 434 | 350 | 1.579 | 0.000 | 0.453 |
| 2,4-diaminobutyric acid 5 | 432 | 200 | 1.551 | 0.000 | 0.224 |
| noradrenaline | 426 | 315 | 1.162 | 0.025 | 0.000 |
| 4-Acetamidobutyric acid 2 | 425 | 174 | 1.616 | 0.000 | 0.000 |
| N-alpha-Acetyl-L-ornithine 1 | 422 | 174 | 1.360 | 0.013 | 366390.125 |
| sorbose 2 | 403 | 330 | 1.615 | 0.000 | 0.000 |
| carbamoyl-aspartic acid 2 | 400 | 355 | 1.427 | 0.001 | 0.001 |
| Dioctyl phthalate | 387 | 319 | 1.162 | 0.030 | 0.000 |
| L-dopa 2 | 384 | 100 | 1.159 | 0.025 | 0.000 |
| 2,3-Dimethylsuccinic acid | 383 | 244 | 1.360 | 0.009 | 289251.427 |
| N(epsilon)-Trimethyllysine | 380 | 96 | 1.166 | 0.026 | 0.000 |
| creatine degr | 380 | 147 | 1.380 | 0.006 | 0.000 |
| N(alpha),N(alpha)-dimethyl-L-histidine | 379 | 174 | 1.608 | 0.000 | 0.015 |
| Phenylphosphoric acid | 373 | 211 | 1.261 | 0.004 | 1.457 |
| aspartic acid 1 | 369 | 210 | 1.078 | 0.012 | 0.665 |
| Adipamide 5 | 344 | 301 | 1.584 | 0.000 | 3.478 |
| 2-ketoadipate 3 | 340 | 96 | 1.197 | 0.006 | 0.799 |
| N-ethylmaleamic acid 3 | 334 | 155 | 1.362 | 0.007 | 2982053.949 |
| 3-hydroxy-3-methylglutaric acid | 334 | 246 | 1.550 | 0.001 | 0.131 |
| D-alanyl-D-alanine 2 | 334 | 99 | 1.121 | 0.032 | 2451131.955 |
| Guanidinosuccinic acid 2 | 324 | 286 | 1.357 | 0.005 | 577465.020 |
| Glycine-d5 | 322 | 370 | 1.118 | 0.009 | 0.442 |
| N-Acetyl-L-glutamic acid 1 | 318 | 290 | 1.160 | 0.027 | 0.000 |
| Benzylsuccinic acid | 317 | 313 | 1.184 | 0.010 | 0.686 |
| 3-hydroxybutyric acid | 301 | 58 | 1.561 | 0.000 | 0.339 |
| Acetol 3 | 299 | 217 | 1.512 | 0.000 | 0.340 |
| 3,5-Dihydroxyphenylglycine 1 | 294 | 283 | 1.216 | 0.009 | 1.582 |
| adrenaline 2 | 290 | 294 | 1.432 | 0.000 | 0.023 |
| panthenol 2 | 289 | 349 | 1.422 | 0.000 | 0.753 |
| 2,8-Dihydroxyquinoline | 283 | 292 | 1.422 | 0.000 | 0.753 |
| 4-hydroxyphenylpyruvate 1 | 282 | 191 | 1.162 | 0.004 | 4.570 |
| hydroquinone | 280 | 270 | 1.576 | 0.000 | 0.205 |
| L-homoserine 1 | 277 | 71 | 1.360 | 0.012 | 347353.442 |
| Isoxanthopterin | 274 | 267 | 1.374 | 0.011 | 0.000 |
| N-cyclohexylformamide 2 | 271 | 392 | 1.062 | 0.038 | 4.628 |
| Catechol | 268 | 195 | 1.203 | 0.018 | 0.433 |
| Vanillylmandelic acid | 268 | 162 | 1.375 | 0.006 | 0.000 |
| Glutaconic acid | 260 | 392 | 1.615 | 0.000 | 0.000 |
| 1,3-Cyclohexanedione 2 | 259 | 180 | 1.242 | 0.004 | 0.802 |
| cis-Phytol | 254 | 86 | 1.241 | 0.004 | 0.706 |
| 1-Hydroxy-2-naphthoic acid | 253 | 283 | 1.314 | 0.003 | 1.968 |
| N-(2-hydroxyethyl)-iminodiacetic acid 1 | 243 | 232 | 1.126 | 0.040 | 106360.593 |
| L-Cysteic acid | 241 | 167 | 1.422 | 0.000 | 0.753 |
| hydroxylamine | 238 | 86 | 1.354 | 0.001 | 1.633 |
| 2-mercaptoethanesulfonic acid 1 | 238 | 89 | 1.133 | 0.037 | 566087.856 |
| cycloleucine 1 | 238 | 255 | 1.404 | 0.000 | 0.432 |
| Dehydroascorbic Acid 2 | 234 | 286 | 1.356 | 0.040 | 0.267 |
| octanal 3 | 231 | 316 | 1.128 | 0.008 | 0.070 |
| 1-Methylhydantoin 1 | 224 | 156 | 1.168 | 0.028 | 0.000 |
| Arginine | 214 | 304 | 1.361 | 0.005 | 250362.698 |
| Cyclohexylamine | 204 | 183 | 1.226 | 0.000 | 13.282 |
| Erythrose 2 | 204 | 96 | 1.155 | 0.016 | 0.310 |
| 3-Methyloxindole 3 | 175 | 200 | 1.422 | 0.000 | 0.753 |
| beta-Hydroxymyristic acid | 167 | 252 | 1.129 | 0.027 | 30866.795 |

Sim represents similarity.
