# Supplementary material for: Metabolomics Responses of Pearl Oysters (Pinctada fucata martensii) Fed a Formulated Diet Indoors and Cultured With Natural Diet Outdoors
Source: Front Physiol. 2018 Jul 19;9:944. doi: 10.3389/fphys.2018.00944 (PMC6060569; doi:10.3389/fphys.2018.00944)
Supplement: Supplementary file 3 [file Table_3.DOCX]

**Supplemental Table 3** Results from the hepatopancreas metabolomics pathway analyses in *P. f. martensii* for group EG vs. CG.

| Pathway | hits | P value | Holm p | Impact |
| --- | --- | --- | --- | --- |
| Cysteine and methionine metabolism | 5 | 0.059622 | 1 | 0.16071 |
| Taurine and hypotaurine metabolism | 2 | 0.083826 | 1 | 0 |
| Sulfur metabolism | 2 | 0.11111 | 1 | 0.33334 |
| Starch and sucrose metabolism | 3 | 0.20969 | 1 | 0.16062 |
| Fructose and mannose metabolism | 3 | 0.23267 | 1 | 0 |
| Glycine, serine and threonine metabolism | 3 | 0.27985 | 1 | 0 |
| Steroid biosynthesis | 1 | 0.29663 | 1 | 0 |
| Phenylalanine, tyrosine and tryptophan biosynthesis | 1 | 0.29663 | 1 | 0 |
| Linoleic acid metabolism | 1 | 0.29663 | 1 | 0 |
| Valine, leucine and isoleucine biosynthesis | 2 | 0.29971 | 1 | 0 |
| Galactose metabolism | 2 | 0.29971 | 1 | 0.03226 |
| Thiamine metabolism | 1 | 0.35603 | 1 | 0 |
| Arginine and proline metabolism | 3 | 0.42342 | 1 | 0.07356 |
| Propanoate metabolism | 2 | 0.48454 | 1 | 0 |
| Butanoate metabolism | 2 | 0.48454 | 1 | 0 |
| Phenylalanine metabolism | 1 | 0.50608 | 1 | 0.30769 |
| Alanine, aspartate and glutamate metabolism | 2 | 0.51252 | 1 | 0 |
| Citrate cycle (TCA cycle) | 2 | 0.51252 | 1 | 0.0975 |
| Glutathione metabolism | 2 | 0.63708 | 1 | 0.01802 |
| Arachidonic acid metabolism | 1 | 0.65374 | 1 | 0 |
| Amino sugar and nucleotide sugar metabolism | 2 | 0.67956 | 1 | 0 |
| Sphingolipid metabolism | 1 | 0.7784 | 1 | 0.02381 |
| Selenoamino acid metabolism | 1 | 0.7784 | 1 | 0.03086 |
| Pyruvate metabolism | 1 | 0.79738 | 1 | 0.1772 |
| Glycerophospholipid metabolism | 1 | 0.88189 | 1 | 0 |
| Tyrosine metabolism | 1 | 0.89209 | 1 | 0 |
| Glycolysis or Gluconeogenesis | 1 | 0.89209 | 1 | 0.09953 |
| Fatty acid elongation in mitochondria | 1 | 0.90995 | 1 | 0 |
| Fatty acid biosynthesis | 1 | 0.9638 | 1 | 0 |
| Valine, leucine and isoleucine degradation | 1 | 0.96698 | 1 | 0 |
| Fatty acid metabolism | 1 | 0.96987 | 1 | 0 |
| Pyrimidine metabolism | 1 | 0.97252 | 1 | 0.05693 |
| Purine metabolism | 1 | 0.99251 | 1 | 0.01564 |
| Aminoacyl-tRNA biosynthesis | 1 | 0.99758 | 1 | 0 |

hits represent the number of metabolites in one pathway;

Home p indicates the statistical P values that were further adjusted using the Holm-Bonferroni method for multiple tests.
